# Complete Genome Sequence of the World Health Organization Mumps Reference Strain, MuVi/Sheffield.GBR/1.05

**DOI:** 10.1128/mra.00336-22

**Published:** 2022-06-07

**Authors:** Jasmine Rae Frost, Alberto Severini

**Affiliations:** a Department of Medical Microbiology and Infectious Diseases, Faculty of Health Sciences, University of Manitoba, Winnipeg, Manitoba, Canada; b JC Wilt Infectious Diseases Research Centre, NMLB, Public Health Agency of Canada, Winnipeg, Manitoba, Canada; DOE Joint Genome Institute

## Abstract

The World Health Organization has designated the MuVi/Sheffield.GBR/1.05 strain as a genotype G mumps reference strain. However, currently only the SH and HN gene sequences are available. We are reporting the complete genome sequence of this strain so that it can now be used as a standard for mumps molecular epidemiology.

## ANNOUNCEMENT

Mumps-like infection was first described by Hippocrates in the 16th century (1, 2). In 1934, Johnson and Goodpasture showed that mumps disease was caused by a virus (3). The mumps RNA virus is part of the *Paramyxoviridae* family, in the genus *Rubulavirus*, and humans are the only host identified (4).

A resurgence of mumps has been identified in young adults and in vaccinated populations (5–10). Current genotyping of the small hydrophobic (SH) gene is insufficient, as current outbreaks are due to genotype G strains with minimal genomic diversity in the SH gene (9, 11–13). As a result, whole-genome sequencing is now used to analyze these outbreaks.

The MuVi/Sheffield.GBR/1.05 G reference strain, as designated by the World Health Organization (WHO), was isolated in the United Kingdom in 2005 and is used to compare mumps SH gene sequences (14). Currently, only the SH and hemagglutinin-neuraminidase (HN) gene sequences of the Sheffield strain are publicly available (14, 15). With the increase in whole-genome sequencing to resolve mumps outbreaks, the complete Sheffield sequence would allow this reference strain to remain consistent across different types of sequence analyses.

The MuVi/Sheffield.GBR/1.05 mumps strain was purchased from the National Collection of Pathogenic Viruses (Salisbury, UK) (catalog number 1604296v) and passaged once in Vero cells (ATTC number CCL-81 [16]). Although the effect of cell culture in introducing mumps mutations is unknown, sequential passaging can select for specific strains (17). Our sample underwent one passage in cell culture; therefore, we expect the effect to be minimal.

Nucleic acid was extracted from 200 μl of sample, and first- and second-strand cDNA syntheses were performed as described previously (18). Libraries were prepared using the TruSeq Nano DNA high-throughput library preparation kit (catalog number 20015965; Illumina), with modifications as described previously (18). The library was sequenced on the MiSeq platform (Illumina) using a MiSeq reagent kit v3 (catalog number MS-102-3003).

Using the Galaxy platform (19), FastQ files were aligned using Bowtie 2 (20) with the settings of pair data set collection and a maximum fragment length of 1,000 bases, with a previously described reference sequence (18). BAM Coverage Statistics was used to determine genomic coverage (21). In total 30,768 reads were mapped in the genomic alignment. The alignment was entered into the SNVPhyl pipeline (minimum mean mapping of 28 and single-nucleotide variant [SNV] abundance ratio of 0.75), and a consensus sequence was constructed using the BCFtools consensus (22, 23). All tools were run with default parameters unless otherwise specified.

The MuVi/Sheffield.GBR/1.05 whole genome was successfully sequenced (including the termini), with a genome size of 15,384 nucleotides and a GC content of 42.3%. The minimum coverage per nucleotide was 4×, the maximum coverage was 8,000×, and the mean coverage per nucleotide was 367.21×.

**FIG 1:**
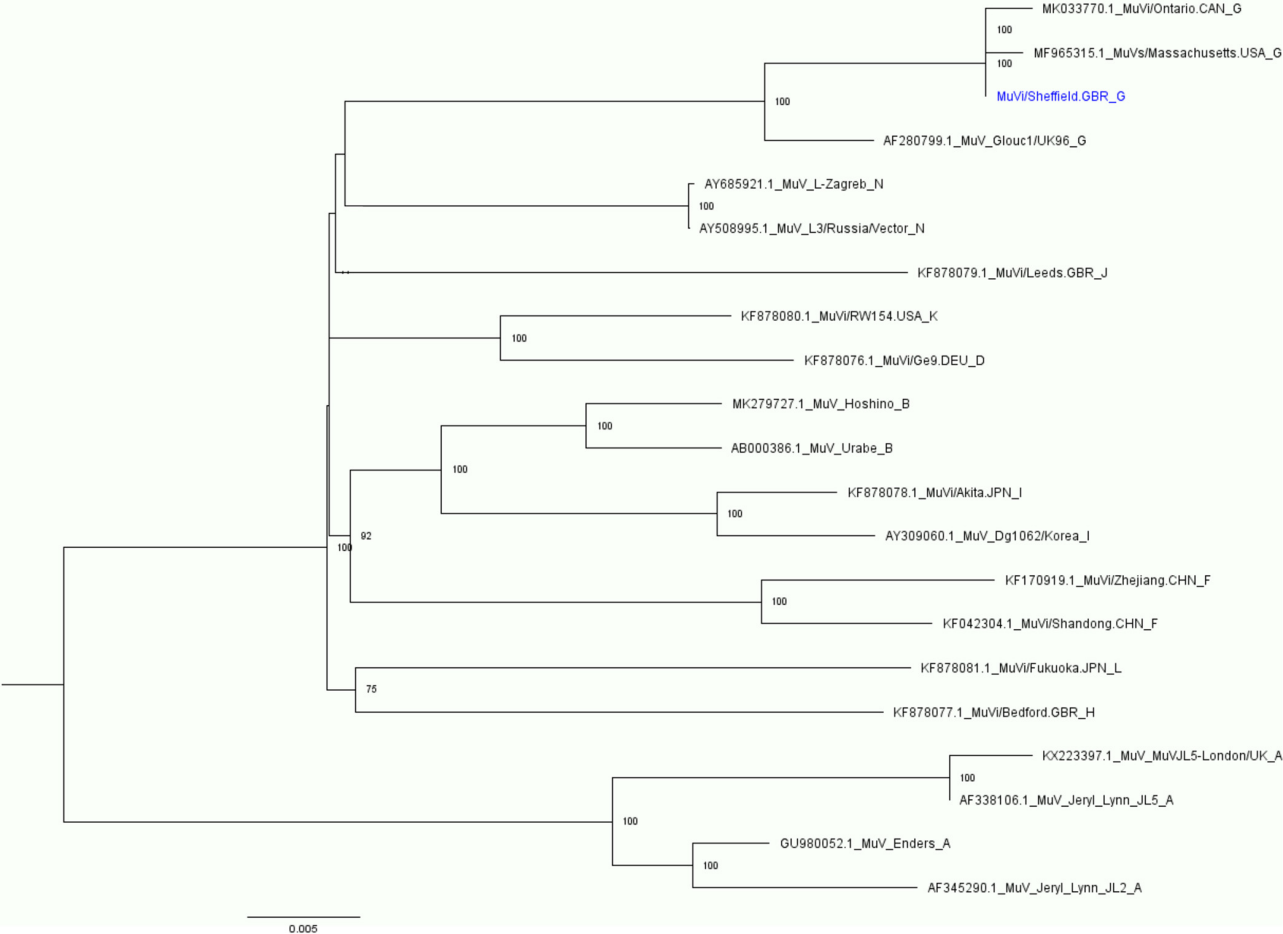
Maximum likelihood tree of published mumps complete genome sequences (accession numbers MK279727.1, MK033770.1, MF965315.1, KX223397.1, KF878081.1, KF878080.1, KF878079.1, KF878078.1, KF878077.1, KF878076.1, KF170919.1, KF042304.1, GU980052.1, AY685921.1, AY508995.1, AY309060.1, AF345290.1, AF338106.1, AF280799.1, and AB000386.1) and our sequenced MuVi/Sheffield.GBR/1.05 (shown in blue). A bootstrap analysis of 1,000 was run, with the percentage indicated at each node (values below 70% were removed). The phylogenetic analysis was done using IQ-TREE 2.1.3 (17).

The Sheffield sequence was analyzed in a maximum likelihood tree with other complete WHO reference strains, vaccine strains, and recent genotype G outbreak strains (Fig. 1). As expected, the MuVi/Sheffield.GBR/1.05 strain clusters with high confidence with outbreak genotype G strains and the alternate WHO genotype G reference strain, MuV Glouc1/UK96 G (Fig. 1).

The MuVi/Sheffield.GBR/1.05 whole-genome sequence adds to the tools available for resolving mumps outbreaks. This will allow for continuity of analysis between SH sequencing and whole-genome sequencing.

### Data availability.

This genome has been submitted to GenBank under accession number ON148331, and the whole-genome sequencing reads have been submitted to SRA (BioProject accession number PRJNA834753).

## References

[1] Hippocrates. 1993. Hippocrates: epidemics I (epidemion to proton). (In Greek.) Athens, Greece.

[2] Mammas IN, Spandidos DA. 2016. Paediatric virology in the Hippocratic Corpus. Exp Ther Med 12:541–549. doi:10.3892/etm.2016.3420. 27446241 PMC4950906

[3] Johnson CD, Goodpasture EW. 1934. An investigation of the etiology of mumps. J Exp Med 59:1–19. doi:10.1084/jem.59.1.1. 19870227 PMC2132344

[4] Rubin S, Eckhaus M, Rennick LJ, Bamford CGG, Duprex WP. 2015. Molecular biology, pathogenesis and pathology of mumps virus. J Pathol 235:242–252. doi:10.1002/path.4445. 25229387 PMC4268314

[5] Dayan GH, Rubin S. 2008. Mumps outbreaks in vaccinated populations: are available mumps vaccines effective enough to prevent outbreaks? Clin Infect Dis 47:1458–1467. doi:10.1086/591196. 18959494

[6] Ferenczi A, Gee S, Cotter S, Kelleher K. 2020. Ongoing mumps outbreak among adolescents and young adults, Ireland, August 2018 to January. Euro Surveill 25:2020. doi:10.2807/1560-7917.ES.2020.25.4.2000047. PMC7001241 32019666

[7] Galazka AM, Robertson SE, Kraigher A, Kraigher A. 1999. Mumps and mumps vaccine: a global review. Bull World Health Organ 77:3–14. 10063655 PMC2557572

[8] Mühlemann K. 2004. The molecular epidemiology of mumps virus. Infect Genet Evol 4:215–219. doi:10.1016/j.meegid.2004.02.003. 15450201

[9] Wohl S, Metsky HC, Schaffner SF, Piantadosi A, Burns M, Lewnard JA, Chak B, Krasilnikova LA, Siddle KJ, Matranga CB, Bankamp B, Hennigan S, Sabina B, Byrne EH, McNall RJ, Shah RR, Qu J, Park DJ, Gharib S, Fitzgerald S, Barreira P, Fleming S, Lett S, Rota PA, Madoff LC, Yozwiak NL, MacInnis BL, Smole S, Grad YH, Sabeti PC. 2020. Combining genomics and epidemiology to track mumps virus transmission in the United States. PLoS Biol 18:e3000611. doi:10.1371/journal.pbio.3000611. 32045407 PMC7012397

[10] Saboui M, Squires SG. 2020. Mumps outbreaks across Canada, 2016 to 2018. Can Commun Dis Rep 46:427–431. doi:10.14745/ccdr.v46i1112a10. 33776589 PMC7986990

[11] Jin L, Örvell C, Myers R, Rota PA, Nakayama T, Forcic D, Hiebert J, Brown KE. 2015. Genomic diversity of mumps virus and global distribution of the 12 genotypes. Rev Med Virol 25:85–101. doi:10.1002/rmv.1819. 25424978

[12] Gouma S, Cremer J, Parkkali S, Veldhuijzen I, van Binnendijk RS, Koopmans MPG. 2016. Mumps virus F gene and HN gene sequencing as a molecular tool to study mumps virus transmission. Infect Genet Evol 45:145–150. doi:10.1016/j.meegid.2016.08.033. 27590714

[13] World Health Organization. 2014. WHO-recommended surveillance standard of mumps. World Health Organization, Geneva, Switzerland.

[14] World Health Organization. 2012. Mumps virus nomenclature update: 2012. Wkly Epidemiol Rec 87:217–224. 24340404

[15] Cui A, Myers R, Xu W, Jin L. 2009. Analysis of the genetic variability of the mumps SH gene in viruses circulating in the UK between 1996 and 2005. Infect Genet Evol 9:71–80. doi:10.1016/j.meegid.2008.10.004. 19007915

[16] Yasumura Y, Kawakita Y. 1963. Studies on SV40 in tissue culture: preliminary step for cancer research in vitro. Nihon Rinsho 21:1201–1215.

[17] Amexis G, Rubin S, Chizhikov V, Pelloquin F, Carbone K, Chumakov K. 2002. Sequence diversity of Jeryl Lynn strain of mumps virus: quantitative mutant analysis for vaccine quality control. Virology 300:171–179. doi:10.1006/viro.2002.1499. 12350348

[18] Frost JR, Schulz H, McLachlan E, Hiebert J, Severini A. 2021. An enrichment method for capturing mumps virus whole genome sequences directly from clinical specimens. J Virol Methods 294:114176. doi:10.1016/j.jviromet.2021.114176. 33957163

[19] Afgan E, Baker D, Batut B, Van Den Beek M, Bouvier D, Ech M, Chilton J, Clements D, Coraor N, Grüning BA, Guerler A, Hillman-Jackson J, Hiltemann S, Jalili V, Rasche H, Soranzo N, Goecks J, Taylor J, Nekrutenko A, Blankenberg D. 2018. The Galaxy platform for accessible, reproducible and collaborative biomedical analyses: 2018 update. Nucleic Acids Res 46:W537–W544. doi:10.1093/nar/gky379. 29790989 PMC6030816

[20] Langmead B, Trapnell C, Pop M, Salzberg SL. 2009. Ultrafast and memory-efficient alignment of short DNA sequences to the human genome. Genome Biol 10:R25. doi:10.1186/gb-2009-10-3-r25. 19261174 PMC2690996

[21] Quinlan AR, Hall IM. 2010. BEDTools: a flexible suite of utilities for comparing genomic features. Bioinformatics 26:841–842. doi:10.1093/bioinformatics/btq033. 20110278 PMC2832824

[22] Petkau A, Mabon P, Sieffert C, Knox NC, Cabral J, Iskander MMM, Iskander MMM, Weedmark K, Zaheer R, Katz LS, Nadon C, Reimer A, Taboada E, Beiko RG, Hsiao W, Brinkman F, Graham M, Van Domselaar G. 2017. SNVPhyl: a single nucleotide variant phylogenomics pipeline for microbial genomic epidemiology. Microb Genom 3:e000116. 29026651 10.1099/mgen.0.000116 PMC5628696

[23] Li H, Handsaker B, Wysoker A, Fennell T, Ruan J, Homer N, Marth G, Abecasis G, Durbin R, 1000 Genome Project Data Processing Subgroup. 2009. The Sequence Alignment/Map format and SAMtools. Bioinformatics 25:2078–2079. doi:10.1093/bioinformatics/btp352. 19505943 PMC2723002

